# *Trans* fatty acid isomers and the *trans*-9/*trans*-11 index in fat containing foods

**DOI:** 10.1002/ejlt.201100037

**Published:** 2011-10

**Authors:** Katrin Kuhnt, Melanie Baehr, Carsten Rohrer, Gerhard Jahreis

**Affiliations:** Department of Nutritional Physiology, Institute of Nutrition, Friedrich Schiller UniversityJena, Germany

**Keywords:** Elaidic acid, *t*9/*t*11 index, *Trans* octadecenoic acids, Vaccenic acid

## Abstract

To determine *trans* fatty acid (TFA) distribution of contemporary foods, especially regarding individual *trans* octadecenoic acids (*trans* C18:1), 339 German foods of six categories (semi-solid fats, deep-fried potato products, bakery products, confectioneries, instant products and butter) were analysed using two GC methods. Results showed a high variation of TFA content between and within the categories containing between 0 and 40.5% of FAME except in butter, which is a source of natural TFA. The mean TFA values were below 2.0% of FAME, however, bakery products contained 4.5% and butter fat 3.2%, respectively. In addition, the distribution of individual *trans* C18:1 differed. In samples containing ruminant fat (butter and various confectioneries), vaccenic acid (*t*11-C18:1, *t*11) predominated, while in foods containing industrially hydrogenated fats, elaidic acid (*trans-*9, *t*9-) and *t*10-C18:1 were the major *trans* isomers.. This was reflected by a low *t*9/*t*11 index of 0.3 and 0.5 in butter and ruminant fat containing confectioneries, respectively, whilst the highest index was observed in shortenings and deep-fried potato products at 5.2 and 6.8, respectively. In conclusion, the TFA content of foods available on the German market is generally declining, but substantial variations are present. The *t*9/*t*11 index could be used as an indicator to determine ruminant fat.

**Practical applications:** A number of studies provide evidence that a high TFA intake, particularly of industrial origin, adversely affects human health. The TFA content of foods could be reduced due to the introduction of several mandatory regulations and modifications regarding the hydrogenation process of oils. The most abundant dietary TFA are the isomers of *trans* C18:1. Unfortunately, the differentiation of these isomers is not yet very common, though the *trans* C18:1 profile differs depending on its origin (bacterial hydrogenation in the rumen or industrial hydrogenation). To date, data for TFA content including the *trans* C18:1 profile of different food categories are limited. The present study confirmed that the TFA contents in German foods are declining. However, TFA are still elevated, especially in bakery products and confectioneries, which are produced using mainly industrial but also ruminant fats. Therefore, the *t*9/*t*11 index imparts important information on the source of TFA in processed foods.

## 1 Introduction

*Trans* fatty acids (TFA) are defined as unsaturated fatty acids containing one or more isolated (i.e. non-conjugated) double bonds in *trans* configuration, excluding the CLA [1]. The two major dietary sources for TFA include production via industrial hydrogenation of vegetable oils (partially hydrogenated vegetable oils, PHVO) and through bacterial hydrogenation in the rumen. Sources of naturally derived TFA (ruminant TFA, R-TFA) are milk, dairy products and meat. Compared to unhydrogenated oils, PHVO containing industrial TFA (I-TFA) are semi-solid, have a higher oxidative stability and a longer shelf life. Globally, PHVO are commonly used in processed food products such as margarines, deep-fried foods, bakery and instant products as well as in confectioneries. Indeed, PHVO in industrially processed foods can contain up to 50% TFA, mainly elaidic acid (*trans-*9-octadecenoic acid, *t*9-C18:1, *t*9) and *t*10-C18:1 (*t*10). In contrast, ruminant fats generally have low quantities of TFA (1–8%), with *t*11-C18:1 (vaccenic acid, *t*11) being the predominant *trans* C18:1 isomer [2, 3].

A number of experimental studies demonstrate a positive correlation between the consumption of I-TFA and the risk of coronary heart disease, due in particular to the adverse influence on the lipoprotein profile in serum [4]. In fact, only a small number of studies have compared the health aspects of an intake of quantitative equal TFA from the two sources, industrial and ruminant (I- and R-TFA). To date, there is no conclusive evidence supporting that moderate consumption of R-TFA (mainly *t*11) is related to adverse physiological effects [5–7]. Epidemiological, clinical and rodent studies suggest that *t*11 intake has no relationship to coronary heart disease or inflammation. Indeed, *t*11 may impart health benefits due to its function as a dietary precursor of *c*9,*t*11-CLA [8, 9].

Estimates of dietary TFA intake differ from country to country. Before the mandatory declaration of TFA content on the nutrition label was introduced in 2003, the intake in the United States (U.S.) and Canada was high compared to most European countries (mean 2.6 en%, percent of energy intake; 5.8 g/day [1]; vs. 0.5–2.1 en%; mean 2.8 g/day [10]). Interestingly, in the U.S., approximately 80% of total TFA intake derives from I-TFA [1]. In contrast, the estimated TFA intake in Germany between 1991 and 1997 ranged from 1.9 to 3.8 g/day [1, 11, 12]. Moreover, the proportion of R-TFA intake in Europe is up to 80% of total TFA [13]. International recommendations regarding the maximum daily TFA intake are scarce. However, there are proposals to keep the TFA intake as low as possible [1, 14]. Due to the detrimental health effects of I-TFA, several countries introduced legislation that enforces a mandatory declaration of TFA content on food labels, for example, U.S. and Canada [1, 15] as well as reducing the proportion of I-TFA in foods. Denmark, Switzerland and Austria authorised that oils and fats in locally made or imported foods must contain less than 2% I-TFA of the total fat content [16–18]. Currently, mandatory legislation regarding TFA content in foods do not exist in Germany and there are no plans for such regulations in the near future, although there are methods available that remove I-TFA from foods without resulting in higher costs, an impaired taste or lower product availability [5]. In general, most data relating to TFA content in foods date back to more than 10 years [19, 12]. Results from current studies reveal a contradictory situation around the globe with TFA-free products on the one hand and foods containing extremely high TFA concentrations on the other [20]. In recent years, alternative fats have been developed that substitute PHVO resulting in a modification of the TFA content in foods. Data from a study conducted in 2005 show that on average the proportion of *trans* C18:1 in various German foods was 2.8% of FAME, although results revealed strong variations from 0 to 55.6% of FAME [3]. Thus, for purposes of obtaining more recent data (2007–2009), a large variety of foods on the German market was analysed in the present study. The data obtained represent the basis for calculating the current TFA intake for Germany's population. The main focus was placed on total fat, saturated fatty acids (SFA) and TFA content, in particular on individual *trans* C18:1 isomers as well as on the *t*9/*t*11 index.

## 2 Materials and methods

### 2.1 Sampling plan

The primary selection of the foods was based on the German National Nutrition Survey II and was related to the contribution of the food items to the total fat intake. A secondary focus was placed on foods considered as high risk foods containing a high TFA content that had been established in previous studies or those considered as having potentially high amounts of processed fats such as PHVO. Between July 2007 and May 2009, processed food items (*n* = 339) were purchased from fast food chains such as McDonald's, and Burger King throughout Germany, from various supermarkets in Thuringia (Aldi, Lidl, Rewe, EDEKA, Kaufland, Netto), and from local Thuringian bakeries and kiosks selling snack-food. Finally, various types of butter were also procured.

### 2.2 Sample classification

The purchased food items available on the German market were classified into six categories: (I) semi-solid fats, *n* = 57; (II) deep-fried potato products, *n* = 61; (III) bakery products, *n* = 60; (IV) confectioneries, *n* = 116; (V) instant products, *n* = 22; (VI) butter samples (*n* = 23). Category (I) was further classified into subgroups of margarines/spreads (*n* = 27) and shortenings/cooking fats (*n* = 30). Category (II) was subclassified into French fries/chips (*n* = 49) and croquettes (*n* = 12). Category (III) was split into puff pastries (palmier, Danish and French pastry; *n* = 37) and doughnuts (e.g. Berliner, bismarck, cruller; *n* = 23), whereas, category (IV) was divided into chocolate products (chocolate bars, drops, candies, ice cups, candy discs and icings; *n* = 31) and biscuits (shortbread, cookies, cream-filled wafers, unfilled and cream-filled biscuits; *n* = 85). Category (V) comprised bouillon cubes, instant soups, instant sauces and stocks. The categories (V) and (VI) were not separated into subgroups.

### 2.3 Pre-analysis

All food samples were lyophilised and homogenised using a laboratory blender. The total fat content was determined by Soxhlet extraction with petroleum ether after acid hydrolysis (Soxtherm; Gerhardt GmbH & Co. KG, Germany). Lipids for fatty acids (FA) analysis were extracted separately from the lyophilised sample according to the method of Folch et al. [21] by means of methanol/chloroform/water mixture at a ratio of 2:1:1 by volume. Due to the low content of free FA (<2%), the lipid extracts (neutral lipids) were transesterified under alkaline conditions with sodium methoxide (NaOCH_3_; 0.5 M in anhydrous methanol at 60°C for 15 min and butter samples at 21°C) to produce FAME.

### 2.4 FA analysis

FAME extracts were analysed by means of GC (equipped with a cooled autosampler and flame ionisation detector). Two different GC procedures were used to analyse FAME distribution [9]. In the first GC method the general FA distribution of FA with chain length containing 4–26 carbons, as well as the total CLA were determined employing a fused-silica capillary column of medium polarity (GC-17 V3 Shimadzu, Kyoto, Japan; DB-225MS: 60 m × 0.25 mm i.d. with 0.25 µm film thickness; Agilent Technologies, USA). Oven temperature was initially maintained for 2 min at 70°C, increased by 10°C/min to 180°C, further increased by 2°C/min to 220°C and held for 5 min. In the final step it was increased by 2°C/min to 230°C and held for 27 min. In the second GC method *cis* and *trans* isomers of C18:1 and of C18:2 *n*-6 were separated using a fused-silica capillary column of high polarity (GC-2010plus, Shimadzu; CP-select 200 m × 0.25 mm i.d. with 0.25 µm film thickness; Varian, The Netherlands). These isomers were separated under isothermal conditions at 176°C. For GC analysis, 1 µL of 2% FAME in *n*-hexane was injected with a split of 1:100. For both procedures the injector and detector temperature was maintained at 260 and 270°C, respectively. The carrier gas was hydrogen [9] and for a complete separation of various isomers of CLA a silver-ion HPLC (LC10A, Shimadzu) was used [22].

### 2.5 Identification and calculation of FAME

The proportion of FAME separated from food lipids was generally expressed as % of total FAME (g/100 g). Total fat content was expressed as % of fresh matter. A total of 85 FA without various CLA isomers were identified, in a minimum area of 500 counts. The total TFA comprised of *t*9-C14:1, *t*9-C16:1, total *trans* C18:1, non-conjugated C18:2 and C18:3 isomers with at least one *trans* double bond, *t*11-C20:1 as well as *t*13-C22:1. The total *trans* C18:1 isomers included *t*4, *t*5, *t*6-8, *t*9, *t*10, *t*11, *t*12, *t*13/14, *t*15 and *t*16. Because GC-analysis with the 200 m polar column was performed without a pre-separation of the *cis* and *trans* C18:1 by Ag^+^-TLC an under- or overestimation of *t*13/14 and *t*15 due to co-elution with the *c*9-C18:1 under isothermal conditions at 176°C is possible. In addition, *c*6-8 and *c*14 could co-elute with *t*13/14 and *t*16, respectively, however, these *cis* C18:1 isomers are negligible [17].

Various reference standards were used as FAME mix to identify FA peaks: No. 463, 674, (Nu-chek Prep, Inc., Elysian, U.S.), BR2, BR4, ME 93 (Larodan, Malmö, Sweden), Supelco® 37 Component FAME Mix, PUFA No. 3, CLA, linoleic-, linolenic- and C18:1 methyl ester mix (Supelco, Bellefonte, USA). LabSolutions software for GC (GCsolution, Shimadzu) was used for peak integration. The separated *cis* and *trans* isomers of C18:1 and C18:2 (*t*/*t*-, *c*/*t*- and *t*/*c*-9,12) were calculated with the sum of the co-eluted peaks of the GC 60 m column.

### 2.6 Statistics

All statistical analyses were performed using SPSS statistics, version 17.0 (©2009 SPSS Statistics 17 Inc, Illinois, USA). *p* ≤ 0.05 indicated significant differences. Results were stated as means with their SD. The difference of means of the subgroups within the respective food category was tested using the *t*-test analysis. Due to different numbers of samples within different subgroups, the multivariate post hoc Scheffé-test was used. For linear regression correlation analysis, the two-sided Pearson correlation coefficient was used.

## 3 Results

**Figure 1:**
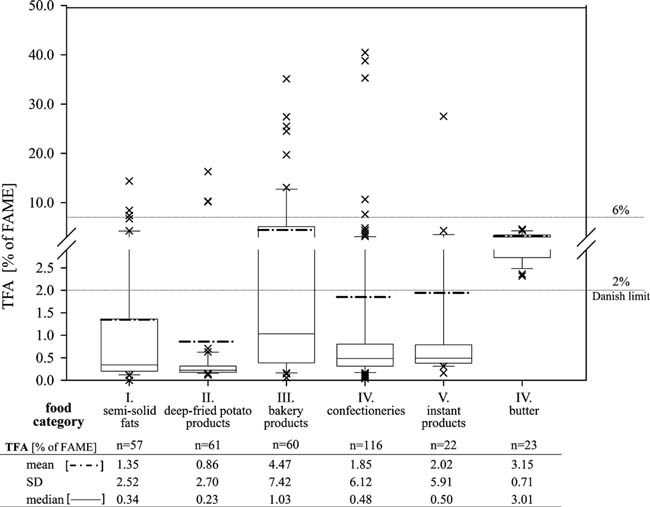
Mean and median TFA proportion of all analysed food categories (% FAME) (––– median, – – – mean, x represents values outside the 10th and 90th percentile).

The mean TFA proportion of all the analysed foods (*n* = 339) independent of the food category was 2.2 ± 5.3% of FAME, however, the median value was 0.47%. In general, TFA content and *trans* C18:1 distribution within the different food categories varied widely. Thus, the median and mean values for TFA differed within all food categories, except for category (VI), the butter samples (Fig. 1). The mean TFA of the food categories was below 2%, except for the categories (III), bakery products and (VI), butter samples, where a TFA content of 4.5 and 3.2%, respectively, was measured (Fig. 1). Furthermore, a comparison of the subgroups in a food category was performed (Table 1).

**Table 1:** FA distribution of foods of the German market (% of FAME); food categories: I. semi-solid fats and II. deep-fried potato products

Food category	(I) Semi-solid fats (*n* = 57)										(II) Deep-fried potato products (*n* = 61)									
		
Subgroup	Margarines/spreads (*n* = 27)					Shortenings/cooking fats (*n* = 30)					French fries/chips (*n* = 49)					Croquettes (*n* = 12)				
				
FA (% of FAME)	Mean	SD	Median	Min	Max	Mean	SD	Median	Min	Max	Mean	SD	Median	Min	Max	Mean	SD	Median	Min	Max
C10:0	0.47	0.45	0.32	0.00	1.55	0.67	1.42	0.10	0.00	6.67	0.02	0.01	0.01	0.00	0.06	0.01	0.03	0.01	0.00	0.12
C12:0	4.30	4.44	2.49	0.08	19.47	5.27	10.38	0.80	0.02	47.51	0.11	0.07	0.11	0.00	0.27	0.16	0.09	0.14	0.02	0.33
C14:0	1.80	1.52	1.36	0.12	6.60	2.50	3.52	1.18	0.07	16.57	0.44	0.36	0.28	0.05	1.02	0.91	0.36	0.95	0.15	1.61
C16:0	18.44^b^	7.30	18.89	6.07	35.35	29.97^a^	13.15	33.76	5.59	48.58	21.06^b^	16.39	15.60	4.15	47.42	38.75^a^	11.80	40.67	9.44	51.10
*c*9-C16:1	0.15	0.09	0.12	0.07	0.52	0.16	0.07	0.15	0.02	0.46	0.15	0.03	0.14	0.09	0.25	0.16	0.04	0.16	0.10	0.23
C18:0	6.30	4.86	4.23	2.96	18.06	5.07	1.92	4.59	2.56	9.61	3.58^b^	0.76	3.21	2.63	4.84	4.71^a^	0.46	4.61	4.01	5.51
*t*4-C18:1	0.00	0.01	0.00	0.00	0.02	0.00	0.00	0.00	0.00	0.02	0.00	0.00	0.00	0.00	0.00	0.00	0.00	0.00	0.00	0.00
*t*5-C18:1	0.00	0.01	0.00	0.00	0.06	0.00	0.01	0.00	0.00	0.02	0.01	0.01	0.00	0.00	0.06	0.00	0.01	0.00	0.00	0.02
*t*6/7/8-C18:1	0.15	0.24	0.01	0.00	0.88	0.26	0.52	0.02	0.00	1.73	0.08	0.25	0.02	0.00	1.65	0.06	0.07	0.02	0.01	0.22
*t*9-C18:1	0.20	0.30	0.03	0.00	1.10	0.51	1.12	0.05	0.00	5.31	0.27	0.80	0.07	0.02	4.12	0.09	0.05	0.06	0.04	0.19
*t*10-C18:1	0.16	0.24	0.02	0.01	0.81	0.31	0.66	0.03	0.00	2.75	0.20	0.64	0.03	0.02	3.67	0.06	0.05	0.03	0.02	0.15
*t*11-C18:1	0.09	0.16	0.00	0.00	0.55	0.15	0.33	0.01	0.00	1.56	0.11	0.41	0.01	0.00	2.25	0.02	0.02	0.01	0.00	0.06
*t*12-C18:1	0.09	0.15	0.00	0.00	0.52	0.11	0.28	0.01	0.00	1.35	0.06	0.22	0.00	0.00	1.24	0.01	0.01	0.00	0.00	0.03
*t*13/14-C18:1	0.06	0.11	0.00	0.00	0.44	0.09	0.19	0.00	0.00	0.79	0.02	0.10	0.00	0.00	0.64	0.01	0.01	0.01	0.00	0.03
*t*15-C18:1	0.14	0.10	0.11	0.04	0.42	0.18	0.21	0.11	0.00	0.94	0.09	0.33	0.02	0.00	1.90	0.02	0.01	0.02	0.01	0.05
*t*16-C18:1	0.00	0.01	0.00	0.00	0.03	0.00	0.01	0.00	0.00	0.04	0.01	0.03	0.00	0.00	0.21	0.00	0.00	0.00	0.00	0.01
*c*9-C18:1	32.38	10.35	27.73	18.48	51.74	34.09	9.02	34.75	6.65	53.40	49.60^a^	14.49	50.29	22.48	69.39	35.12^b^	3.60	35.12	26.90	40.63
*c*11-C18:1	1.32	0.69	1.12	0.53	2.83	1.24	0.66	1.30	0.11	3.28	1.44^a^	0.73	1.45	0.69	3.58	0.75^b^	0.11	0.79	0.53	0.88
*c*12-C18:1	0.05	0.08	0.01	0.00	0.30	0.20	0.80	0.00	0.00	4.39	0.13	0.53	0.00	0.00	2.64	0.00	0.01	0.00	0.00	0.03
*c*9,12-C18:2	29.11^a^	13.70	30.32	10.71	48.49	14.80^b^	11.56	10.38	1.65	45.87	19.07	12.40	17.29	5.69	60.83	17.56	14.48	12.46	6.25	56.28
*c*9,12,15-C18:3	2.32	2.15	2.42	0.07	7.59	1.83	1.77	1.99	0.03	6.90	1.54^a^	1.49	1.44	0.21	5.16	0.28^b^	0.08	0.26	0.18	0.46
C20:0	0.42	0.15	0.35	0.26	0.85	0.43	0.12	0.44	0.09	0.81	0.41	0.07	0.40	0.29	0.67	0.40	0.05	0.40	0.32	0.49
*c*11-C20:1	0.44	0.32	0.31	0.12	1.09	0.39	0.29	0.41	0.05	1.13	0.43	0.29	0.51	0.14	1.26	0.14	0.02	0.14	0.11	0.19
C22:0	0.36	0.17	0.34	0.12	0.71	0.22	0.25	0.15	0.02	1.33	0.44	0.30	0.35	0.07	0.89	0.22	0.20	0.12	0.07	0.74
∑SFA	33.03^b^	8.30	33.05	20.10	55.31	45.36^a^	17.23	45.36	11.05	91.49	26.35^b^	17.15	20.47	8.33	53.93	45.42^a^	12.39	46.79	15.30	59.56
∑MUFA	34.51	11.46	29.55	19.91	55.64	36.27	10.31	36.47	6.83	63.62	52.78^a^	15.60	57.15	24.69	73.74	36.49^b^	3.82	36.47	27.99	42.45
∑PUFA	31.55^a^	12.62	32.93	12.27	52.82	16.78^b^	12.66	13.13	1.68	52.40	20.87	13.19	20.62	8.00	61.42	18.09	14.43	12.79	7.12	56.70
∑*n*-3	0.00	0.01	0.00	0.00	0.02	0.00	0.00	0.00	0.00	0.02	1.65	1.49	1.56	0.23	5.19	0.30	0.09	0.29	0.19	0.51
∑*n*-6	29.13^a^	13.68	30.36	10.72	48.49	14.82^b^	11.58	10.41	1.65	45.91	19.39	12.20	17.29	8.10	60.89	17.61	14.49	12.49	6.36	56.35
∑CLA	0.04	0.01	0.03	0.01	0.07	0.03	0.05	0.02	0.00	0.26	0.14	0.10	0.11	0.03	0.75	0.23	0.13	0.21	0.05	0.51
∑TFAa)	0.96	1.26	0.34	0.11	4.28	1.70	3.26	0.34	0.05	14.38	0.99	3.00	0.22	0.12	16.30	0.32	0.20	0.24	0.13	0.71
∑*Trans* C18:2a)	0.00	0.00	0.00	0.00	0.00	0.00	0.01	0.00	0.00	0.02	0.09	0.38	0.01	0.00	2.66	0.02	0.02	0.02	0.00	0.07
Total fat (% FM)	70.63	17.49	80.00	24.00	80.00	87.80	10.55	80.00	74.00	100.00	11.7^a^	4.8	12.0	4.2	19.4	8.9^b^	3.7	8.3	4.8	18.2
Portion of foods (%)																				
Between 2 and 6% TFA			22					7					0					0		
Above 6% TFA			0					13					6					0		

Food categories: (I) semi-solid fats; (II) deep-fried potato products; (III) bakery products; (IV) confectioneries; (V) instant products; (VI) butter.

^a,b,c^Mean values with different superscript letters were significantly different between subgroups of one food category (*p* ≤ 0.05).

a) Isomeres with at least one isolated *trans*-double bond, no CLA included.

**Table 1a:** food categories: III. bakery products and IV. confectioneries

Food category	(III) Bakery products (*n* = 60)										(IV) Confectioneries (*n* = 116)									
		
Subgroup	Puff pastries (*n* = 37)					Doughnuts (*n* = 23)					Chocolate productsb) (*n* = 31)					Biscuitsc) (*n* = 85)				
				
FA (% of FAME)	Mean	SD	Median	Min	Max	Mean	SD	Median	Min	Max	Mean	SD	Median	Min	Max	Mean	SD	Median	Min	Max
C10:0	0.21	0.34	0.05	0.00	1.65	0.16	0.23	0.05	0.00	0.74	0.61	0.76	0.30	0.00	3.11	0.54	0.76	0.24	0.00	2.40
C12:0	2.53	3.26	0.69	0.07	12.61	1.24	1.80	0.46	0.12	7.45	12.85^a^	15.29	4.14	0.11	50.58	6.89^b^	13.13	0.61	0.20	46.79
C14:0	2.03	1.93	1.16	0.63	11.11	1.33	0.82	0.99	0.42	3.65	6.68^a^	6.04	4.73	0.35	21.14	4.19^b^	5.30	2.00	0.70	19.99
C16:0	40.37^a^	7.16	39.00	31.37	66.24	32.57^b^	9.72	36.30	14.64	45.42	27.51	10.80	27.77	9.49	48.13	28.64	7.41	29.81	11.68	40.96
*c*9-C16:1	0.23	0.26	0.15	0.10	1.63	0.35	0.12	0.33	0.18	0.69	0.33	0.41	0.20	0.00	1.86	0.36	0.13	0.35	0.02	0.58
C18:0	7.71^a^	2.90	7.42	4.19	15.61	6.03^b^	1.37	5.70	4.17	8.79	13.26^b^	6.99	12.32	3.88	34.71	20.58^a^	6.85	20.00	6.39	33.03
t4-C18:1	0.00	0.01	0.00	0.00	0.02	0.00	0.01	0.00	0.00	0.07	0.00	0.01	0.00	0.00	0.07	0.00	0.00	0.00	0.00	0.00
*t*5-C18:1	0.01	0.02	0.00	0.00	0.09	0.03	0.06	0.00	0.00	0.21	0.01	0.03	0.00	0.00	0.21	0.01	0.04	0.00	0.00	0.22
*t*6/7/8-C18:1	0.49	0.57	0.13	0.00	1.94	1.46	2.23	0.26	0.00	7.32	0.37	1.52	0.06	0.00	8.52	0.25	0.78	0.03	0.00	3.82
*t*9-C18:1	0.86	1.03	0.18	0.00	3.37	2.07	3.02	0.38	0.04	9.65	0.44	1.71	0.07	0.00	10.24	0.18	0.45	0.06	0.00	2.08
*t*10-C18:1	0.50	0.55	0.17	0.00	1.88	1.35	2.02	0.24	0.02	6.48	0.34	1.24	0.07	0.00	7.10	0.16	0.36	0.05	0.00	1.64
*t*11-C18:1	0.22	0.24	0.09	0.00	0.85	0.66	1.01	0.15	0.00	2.83	0.27	0.66	0.07	0.00	3.46	0.16	0.17	0.10	0.02	0.76
*t*12-C18:1	0.17	0.20	0.05	0.00	0.68	0.55	0.88	0.12	0.00	2.56	0.16	0.57	0.03	0.00	3.20	0.07	0.12	0.04	0.00	0.67
*t*13/14-C18:1	0.14	0.17	0.03	0.00	0.56	0.46	0.78	0.03	0.00	2.54	0.14	0.56	0.00	0.00	3.14	0.06	0.16	0.02	0.00	0.82
*t*15-C18:1	0.17	0.17	0.06	0.00	0.62	0.46	0.69	0.13	0.00	2.26	0.17	0.48	0.04	0.01	2.79	0.07	0.11	0.04	0.00	0.56
*t*16-C18:1	0.02	0.06	0.00	0.00	0.38	0.05	0.11	0.00	0.00	0.40	0.05	0.12	0.00	0.00	0.48	0.05	0.03	0.04	0.00	0.11
*c*9-C18:1	30.99^b^	8.10	32.94	1.70	41.56	36.37^a^	6.22	37.14	22.87	46.82	28.12	11.34	29.23	1.03	57.90	29.23	9.29	31.72	4.97	48.00
*c*11-C18:1	1.02	0.32	1.05	0.06	1.58	1.30	0.45	1.14	0.69	2.21	0.65^a^	0.42	0.60	0.04	2.48	0.49^b^	0.13	0.51	0.16	0.76
*c*12-C18:1	0.17	0.22	0.05	0.00	0.71	0.39	0.61	0.04	0.00	1.84	0.08	0.24	0.02	0.00	1.33	0.03	0.03	0.03	0.00	0.12
*c*9,12-C18:2	9.16	2.25	9.13	2.79	15.85	9.43	2.87	9.69	2.74	13.89	5.99	3.54	5.36	0.46	23.11	5.63	4.41	4.11	0.44	16.97
*c*9,12,15-C18:3	1.22^a^	0.55	1.21	0.18	2.52	0.84^b^	0.80	0.47	0.13	3.30	0.27	0.28	0.21	0.03	1.72	0.25	0.14	0.25	0.05	0.90
C20:0	0.47	0.12	0.42	0.22	0.83	0.52	0.23	0.42	0.28	1.13	0.44	0.21	0.40	0.15	1.00	0.73	0.22	0.79	0.14	1.01
*c*11-C20:1	0.29	0.10	0.30	0.09	0.51	0.37	0.22	0.30	0.16	0.84	0.11	0.07	0.10	0.00	0.38	0.17	0.25	0.07	0.00	0.94
C22:0	0.14	0.05	0.13	0.07	0.27	0.52	0.78	0.14	0.00	2.44	0.10^b^	0.06	0.09	0.00	0.38	0.27^a^	0.40	0.13	0.04	1.46
∑SFA	53.98^a^	8.23	52.17	42.59	81.50	43.15^b^	7.71	45.25	29.75	56.65	62.04	15.38	60.33	26.93	98.12	62.47	11.92	61.47	42.35	90.07
∑MUFA	35.49^b^	8.81	36.83	2.83	45.15	46.25^a^	8.38	43.44	34.00	62.61	31.52	13.18	32.52	1.38	68.32	31.49	8.80	33.41	6.97	50.67
∑PUFA	10.52	2.38	10.47	4.92	18.18	10.54	3.29	11.08	3.64	17.37	6.45	3.70	6.00	0.49	24.64	6.04	4.40	4.52	0.62	17.25
∑*n*-3	1.39	0.55	1.34	0.52	2.56	1.30	0.93	1.17	0.27	3.47	0.45	0.58	0.26	0.05	3.11	0.32	0.16	0.29	0.10	0.95
∑*n*-6	9.56	2.11	9.36	3.94	15.88	10.54	1.99	10.77	6.84	13.98	6.30	3.54	6.02	0.48	23.23	5.78	4.35	4.25	1.32	17.04
∑CLA	0.07	0.12	0.04	0.02	0.72	0.10	0.05	0.09	0.04	0.23	0.11	0.19	0.06	0.00	1.21	0.10	0.06	0.09	0.00	0.32
∑TFAa)	2.69	2.87	0.80	0.06	9.96	7.34^†^	10.97	1.44	0.14	35.11	2.11	7.02	0.48	0.05	40.46	1.13	2.20	0.52	0.04	10.66
∑*Trans* C18:2a)	0.03	0.08	0.01	0.00	0.45	0.06	0.10	0.01	0.00	0.29	0.06	0.13	0.00	0.00	0.59	0.05	0.04	0.03	0.00	0.14
Total fat (% FM)	26.0^a^	5.0	26.0	16.2	35.1	18.0^b^	6.6	17.6	11.0	37.6	26.7	7.9	27.0	0.9	47.5	24.3	6.1	24.6	11.4	36.2
Portion of foods (%)																				
Between 2 and 6% TFA			32					9					0					8		
Above 6% TFA			11					30					7					4		

^a,b,c^Mean values with different superscript letters were significantly different between subgroups of one food category (*p* ≤ 0.05; ^†^*p* ≤ 0.1).

a) Isomeres with at least one isolated *trans*-double bond, no CLA included.

b) Chocolate bars, drops, candies, ice cups, candy discs and icings.

c) Shortbread, cookies, cream-filled wafers, unfilled and cream-filled biscuits.

**Table 1b:** food categories: V. instant products and VI. butter samples

Food category	(V) Instant products (*n* = 22)					(VI) Butter (*n* = 23)				
		
FA (% of FAME)	Mean	SD	Median	Min	Max	Mean	SD	Median	Min	Max
C10:0	0.26	1.09	0.02	0.00	5.03	Σ ≤ C6 6.60	0.68	6.72	4.75	7.32
C12:0	2.29	9.55	0.22	0.07	43.96	ΣC7-12 8.76	1.03	8.82	5.80	10.39
C14:0	1.68	3.21	1.01	0.33	15.53	10.24	1.05	10.38	6.98	11.40
C16:0	41.71	15.05	43.62	11.44	68.52	27.06	2.17	27.52	22.35	29.99
*c*9-C16:1	0.34	0.49	0.15	0.07	2.07	1.75	0.20	1.77	1.37	1.99
C18:0	8.01	7.17	4.83	3.18	32.21	9.16	0.74	9.12	7.52	10.29
*t*4-C18:1	0.00	0.02	0.00	0.00	0.08	0.02	0.01	0.02	0.00	0.03
*t*5-C18:1	0.01	0.03	0.00	0.00	0.16	0.01	0.01	0.01	0.00	0.03
*t*6/7/8-C18:1	0.27	0.88	0.03	0.01	4.00	0.16	0.05	0.14	0.11	0.29
*t*9-C18:1	0.36	0.97	0.08	0.04	4.50	0.23	0.04	0.22	0.18	0.33
*t*10-C18:1	0.35	1.00	0.08	0.04	4.62	0.27	0.11	0.22	0.17	0.48
*t*11-C18:1	0.27	0.94	0.03	0.01	4.34	1.04	0.57	0.85	0.50	2.32
*t*12-C18:1	0.22	0.84	0.00	0.00	3.87	0.24	0.06	0.22	0.19	0.39
*t*13/14-C18:1	0.13	0.47	0.00	0.00	2.16	0.28	0.05	0.29	0.18	0.35
*t*15-C18:1	0.09	0.41	0.00	0.00	1.89	0.17	0.04	0.17	0.11	0.23
*t*16-C18:1	0.04	0.19	0.00	0.00	0.86	0.31	0.05	0.29	0.21	0.40
*c*9-C18:1	31.84	12.58	32.17	3.85	61.34	20.27	3.05	19.71	16.92	29.94
*c*11-C18:1	1.02	0.66	0.87	0.10	2.79	0.66	0.23	0.58	0.39	1.20
*c*12-C18:1	0.06	0.15	0.00	0.00	0.66	0.17	0.06	0.16	0.07	0.28
*c*9,12-C18:2	0.08	0.05	0.08	0.00	0.19	1.84	1.39	1.54	0.83	6.36
*c*9,12,15-C18:3	0.61	0.74	0.23	0.04	2.76	0.67	0.63	0.50	0.37	2.73
C20:0	0.36	0.13	0.34	0.19	0.84	0.16	0.04	0.15	0.13	0.30
*c*11-C20:1	0.26	0.55	0.13	0.00	2.64	0.08	0.11	0.05	0.04	0.43
C22:0	0.10	0.08	0.08	0.04	0.39	0.07	0.03	0.06	0.05	0.15
∑SFA	55.09	16.55	55.87	20.71	94.96	67.80	4.80	68.79	53.03	73.86
∑MUFA	35.44	13.40	33.70	4.56	65.94	24.86	3.17	24.12	21.15	34.33
∑PUFA	9.47	5.11	8.27	0.48	24.81	3.19	1.95	2.61	2.00	9.46
∑*n*-3	0.73	0.83	0.32	0.05	2.76	1.06	0.50	0.88	0.57	2.16
∑*n*-6	9.13	4.56	8.25	0.49	23.67	0.88	0.65	0.73	0.09	2.89
∑CLA	0.01	0.01	0.00	0.00	0.02	1.06	0.50	0.88	0.57	2.16
∑TFAa)	2.02	5.91	0.50	0.16	27.52	3.15	0.71	3.01	2.32	4.63
∑*Trans* C18:2a)	0.20	0.21	0.19	0.01	1.00	0.37	0.06	0.38	0.21	0.47
Total fat (% FM)	14.00	9.90	10.33	2.32	27.30	Not analysed; minimum is 82% milk fat				
Portion of foods (%)										
Between 2 and 6% TFA			5					100		
Above 6% TFA			5					0		

a) Isomeres with at least one isolated *trans*-double bond, no CLA included.

### 3.1 Food category I: Semi-solid fats

The mean total fat content in this category (*n* = 57) was 79.7 ± 16.6%. The mean TFA content was 1.4 ± 2.5% of FAME, while median TFA content was 0.34% (Fig. 1).

In the subgroup of margarines/spreads (*n* = 27), a lower SFA (mainly C16:0) and a higher PUFA (mainly *n*-6 PUFA) content was seen compared to the subgroup of shortenings/cooking fats (*n* = 30; Table 1). However, TFA proportion was similar in both subgroups (0.96 ± 1.3 and 1.7 ± 3.3%). For 22% of margarines, a value for TFA content between 2 and 6% was found (Table 1). Whilst none of the margarine samples had a TFA content higher than 6%, in the subgroup of shortenings, 13% of the samples had higher TFA values (Table 1).

Splitting up the semi-solid fats into industrial and household fats (*n* = 15 and 42, respectively), there was no significant difference between TFA contents due to the high TFA variation (1.1 ± 2.4% vs. 2.0 ± 2.8%; *p* = 0.234, data not shown). The TFA content in semi-solid fats for household (margarines; *n* = 27 vs. shortenings; *n* = 15) showed no significant differences for the fat type (TFA: margarines: 0.96 ± 1.3% vs. shortenings: 1.4 ± 3.6%; *p* = 0.550).

### 3.2 Food category II: Deep-fried potato products

The mean fat content of all deep-fried potato products (*n* = 61) was 11.2 ± 4.9% with MUFA as major FA group (49.6 ± 14.3% of FAME). The mean TFA content was 0.86 ± 2.7% whilst the median TFA value was 0.23% (Fig. 1).

In the subgroups French fries/chips (*n* = 49) and croquettes (*n* = 12) no significant differences in the TFA content were observed (0.99 ± 3.0% vs. 0.32 ± 0.20%). Six percent of the analysed French fries had a TFA content over 6% (Table 1). Differences in total fat content relating to origin and manufacturing process were not found (data not shown). Deep-frozen discounter products for heating up in the oven (*n* = 27) contained only half the total fat content found in deep-fried products from snack bars (*n* = 34; 7.4 ± 3.2% vs. 15.1 ± 2.7%; *p* = 0.001). However, discounter products had notably higher SFA proportions than those from snack bars (42.8 ± 10.7% vs. 14.8 ± 10.6%; *p* = 0.001). On the other hand, TFA content in discounter products (0.25 ± 0.15%) and in products from fast food chains, such as McDonald's and Burger King sampled in Germany (0.22 ± 0.07%) was low. For French fries samples only two local snack bars featured repeatedly high TFA proportions. In foods with no labelling regarding the type of fat, the analysed TFA content was significantly higher compared to foods with a declared use of vegetable oil, vegetable fat or unhardened fat on the food label (5.5 ± 7.4% vs. 0.24 ± 0.12%, 0.28 ± 0.16% and 0.17 ± 0.04%; *p* ≤ 0.05).

### 3.3 Food category III: Bakery products

The mean fat content of this category (*n* = 60) was 22.9 ± 6.9%. The major FA group involved the SFA group with 49.8 ± 8.9% of FAME and mean TFA content was 4.5 ± 7.4% with a median of 1.0% (Fig. 1).

Comparing the subgroup of puff pastries with doughnuts revealed that puff pastries (*n* = 37) had a higher fat, but lower TFA contents (*n* = 23; Table 1). However, the difference in TFA content was not significant, due to the wide variation of TFA proportions, despite the product similarity within each subgroup (2.7 ± 2.9% vs. 7.3 ± 11.0%; *p* ≤ 0.1). The products from local bakeries generally had twice the TFA content of the discounter products (5.3 ± 8.4% vs. 2.3 ± 2.4%; *p* ≤ 0.05, data not shown).

### 3.4 Food category IV: Confectioneries

The mean fat content of foods in this category (*n* = 116) amounted to 26.1 ± 7.5%. The SFA were the major FA group with 62.2 ± 14.5% of FAME. The mean TFA content was 1.9 ± 6.1% with a median of 0.48% (Fig. 1).

The proportion of SFA did not differ between the two subgroups: chocolate products and biscuits. However, in chocolate products, higher proportions of single SFA such as C12:0 and C14:0 and lower fractions of C18:0 were observed. Due to a high variation in the subgroup of biscuits, the TFA content did not differ significantly (2.1 ± 7.0% vs. 1.1 ± 2.2%; Table 1). Further subdivision of biscuits into cream-filled wafers, unfilled and cream-filled biscuits, demonstrated the highest total fat and TFA contents for cream-filled wafers. Once more, the difference in TFA contents was without statistical significance due to a high variation (Table 2). Interestingly, according to the list of ingredients, confectioneries containing ruminant fat, such as cream, butter and/or milk, had a significantly higher CLA content similar to the subgroup of unfilled biscuits (Table 2).

**Table 2:** Total fat (% fresh matter), TFA and CLA contents (% of FAME) of subgroups of the confectioneries

	Confectioneries (*n* = 116)			
	
	Biscuits (*n* = 85)			
		
Product type	Cream-filled biscuits (*n* = 19)	Unfilled biscuits (*n* = 13)	Cream-filled wafers (*n* = 53)	Chocolate products (*n* = 31)
Total fat (% of fresh matter)	21.6 ± 4.6^b^	21.0 ± 7.0^b^	29.9 ± 7.4^a^	24.3 ± 6.1^b^
TFA (% of FAME)	0.68 ± 0.39	2.0 ± 1.8	2.7 ± 8.8	1.1 ± 2.2
CLA (% of FAME)	0.10 ± 0.08^b^	0.37 ± 0.36^a^	0.06 ± 0.04^b^	0.11 ± 0.06^b^

	Confectioneries (*n* = 116)	
	
	Without ruminant fat (*n* = 56)	With a portion of ruminant fata) (*n* = 60)
Total fat (% of fresh matter)	26.6 ± 8.3	25.6 ± 6.7
TFA (% of FAME)	2.4 ± 8.0b)	1.1 ± 1.7b)
CLA (% of FAME)	0.01 ± 0.00^b^	0.21 ± 0.19^a^
		Confectioneries with exclusively butter fat, *n* = 6; CLA: 0.72 ± 0.34^c^

Mean ± SD.

^a,b,c^Means with different superscripts within a row were significantly different (Scheffé test; *p* ≤ 0.05).

**Figure 2:**
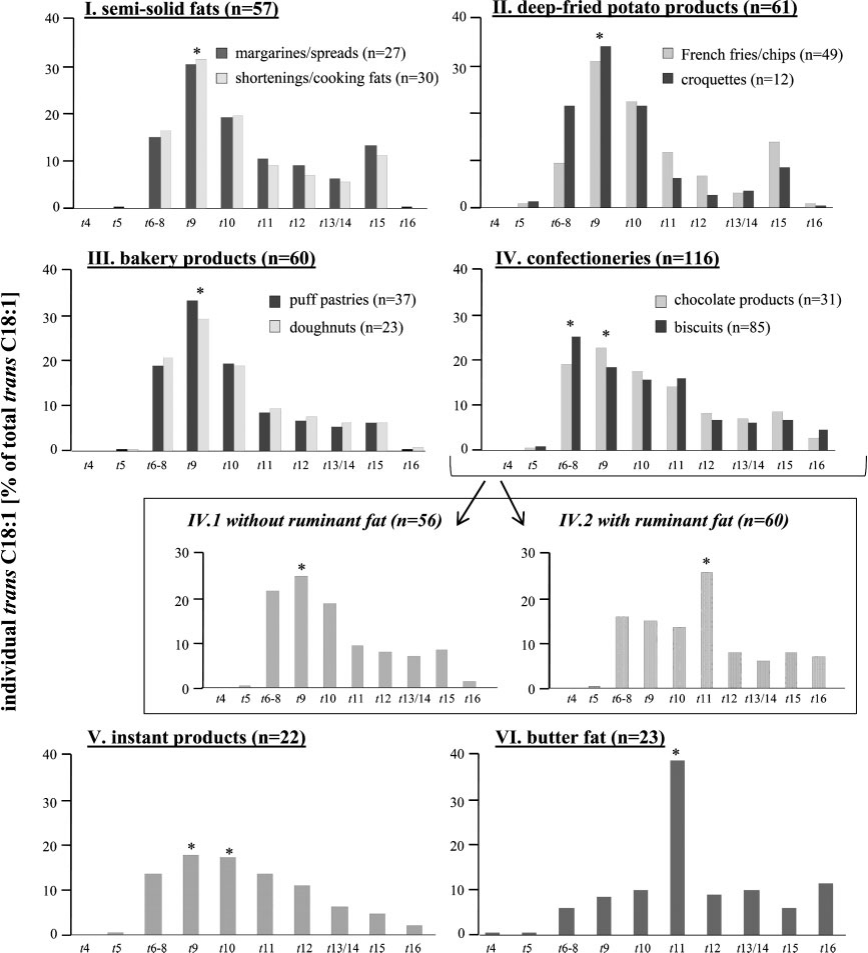
The individual *trans* C18:1 distribution of different food categories and their subgroups (% of total *trans* C18:1). *Significantly different to the other individual *trans* C18:1 isomers with no asterisk within a subgroup (*p* ≤ 0.05).

a) With any portion of milk, butter and/or cream.

b) TFA with different pattern of individual *trans* C18:1 isomers (see Figure 2).

### 3.5 Food category V: Instant products

The mean fat content of instant products of 14.0 ± 9.9% varied widely between 2.3 and 27.3% (Table 1). SFA was the major FA group (55.1% of FAME) and the TFA content amounted to 2.0 ± 5.9% with high variation from 0.16 to 27.5%.

### 3.6 Food category VI: Butter samples

The distribution of FA in unprocessed butter samples (*n* = 23) with fat of only ruminant origin was compared to the FA distribution in industrially processed foods containing fats of different origin. The mean proportion of SFA was 67.8 ± 4.8% of FAME composed mainly of C16:0 followed by short chain and medium chain FA (Table 1). The mean TFA content was 3.2 ± 0.71% and the median was 3.0%. The average CLA content was found to be 1.1 ± 0.50%. The quantity of the most abundant CLA isomer *c*9,*t*11-CLA (0.87 ± 0.35%) positively correlated with the proportion of the major *trans* C18:1 isomer *t*11 (1.0 ± 0.57%; *y* = 0.769*x* + 0.079; *R*^2^ = 0.992; *p* ≤ 0.001).

### 3.7 *Trans* C18:1 profile and *t*9/*t*11 index of foods

The *trans* C18:1 profiles of subgroups within the same food category were similar (Fig. 2). In all subgroups, except for butter, *t*9 was generally the major *trans* C18:1 isomer (ca. 30% of total *trans* C18:1). Moreover, the sum of *t*9 and *t*10 was approximately 50% of the *trans* C18:1. In contrast, in confectioneries containing a proportion of ruminant fat such as milk fat, butter or cream (*n* = 60), and in pure butter fat, *t*11 was the predominant *trans* C18:1 isomer (Fig. 2).

Calculation of the *t*9/*t*11 index resulted in the lowest value for butter fat at 0.3, since *t*11 was continuously higher compared to *t*9 (Table 3). Of all industrially processed foods, French fries/chips showed the highest index compared to confectioneries containing ruminant fat (6.8 vs. 0.5; Table 3).

**Table 3:** The *t*9/*t*11 index of the food categories and subgroups

Food category	Subgroup	*t*9/*t*11 Index (mean)
(I) Semi-solid fats (*n* = 57)	Margarines/spreads (*n* = 27)	2.6
	Shortenings/cooking fats (*n* = 30)	5.2
(II) Deep-fried potato products (*n* = 61)	French fries/chips (*n* = 49)	6.8
	Croquettes (*n* = 12)	6.5
(III) Bakery products (*n* = 60)	Puff pastries (*n* = 37)	3.9
	Doughnuts (*n* = 23)	4.5
(IV) Confectioneries (*n* = 116)	Chocolate productsa) (*n* = 31)	1.2
	Biscuitsb) (*n* = 85)	1.7
(IV) 1	Without ruminant fat (*n* = 56)	2.7
(IV) 2	With ruminant fat (*n* = 60)	0.5
(V) Instant products (*n* = 22)		2.9
(VI) Butter (*n* = 23)		0.3

a) Chocolate bars, drops, candies, ice cups, candy discs and icings.

b) Shortbread, cookies, cream-filled wafers, unfilled and cream-filled biscuits.

## 4 Discussion

### 4.1 Declining trend of TFA content in foods

Comparing the present results with data from prior food studies was challenging due to the heterogeneity of the methods and the varying number of food items analysed. Moreover, in the majority of the previous studies only the sum of all TFA was determined, without differentiating between single *trans* isomers. On average, assessing the present data with previous findings revealed declining TFA contents in the analysed foods. In the present study, 96% of the deep-fried potato products, 90% of the confectioneries, 90% of the instant products and 82% of the semi-solid fats contained less than 2% TFA of FAME (Table 1). In contrast, in Germany average TFA content in 1992 for *semi-solid fats* (margarines/shortenings, *n* = 21) was 9.5% [19]. This value subsequently declined to 1.5% in 1997 [12], a figure which is consistent with present data (1.4%).

In the 1990s, the mean TFA content of *deep-fried potato products* from fast-food restaurants and pre-fried originating from different countries at 16% was also high [23]. In the same period, German French fries samples had approximately 20% TFA [23]. In contrast, in the present study, the TFA content in German deep-fried potato products measured 0.99%. The TFA content of French fries samples from different fast food restaurants (McDonald's and Burger King) in Germany has been dropping steadily (0.22 ± 0.07%, *n* = 25), most likely due to an extensive self-monitoring process by international fast food chains as a result of press releases regarding TFA impact on coronary heart disease. Today, the TFA proportion in deep-fried potato products can be lowered by using frying oils low in TFA. In contrast, during a period of 6 months, high TFA contents were observed repeatedly in French fries from local unaffiliated snack bars in Thuringia (e.g. 15.2 and 12.3%).

In the present study the mean TFA value in 116 *confectioneries* was below 2%, although values up to 41% were found (Fig. 1). In confectioneries of the Swiss market the mean TFA content was 0.80% with contents up to 20% [17]. However, comparisons in this category are generally difficult since studies dealing with TFA in confectioneries are limited [17, 24].

The category of *bakery products* had the highest average TFA content (4.5%). Forty percent of all the 60 bakery products showed TFA contents >2%. In fact, 7% had extremely high TFA proportions (>20%). In Switzerland, Richter et al. [17] sampled bakery products (*n* = 31) containing on average 6.1% TFA and in Austria, Lehner [25] reported that 63% of the analysed bakery products had TFA proportions >2%. Thus, a considerable amount of the TFA intake in the above-mentioned populations can be attributed to bakery products.

### 4.2 High variation of TFA contents in foods

In previous analyses, high variations in the TFA content in predominantly industrially processed foods were observed. In Germany, two studies revealed that TFA in semi-solid fats and bakery products varied from 0 to 24% and from 0 to 16%, respectively, [19, 12]. In another German study, the amount of *trans* C18:1 of 725 different foods also varied highly between 0 and 56% with divergent mean and median values of 2.8 and 0.6%, respectively, [3]. The present study shows similar results with mean and median TFA contents of all foods of 2.2 and 0.47%. TFA contents, particularly in bakery products, varied highly from 0 to 35% and in confectioneries from 0 to 40%, respectively (Table 1). In addition to this variability within the categories, results of the TRANSFAIR study and analysis from Stender et al. [20] indicate a high variation of TFA contents between food items from different countries. In food samples from West European countries, the proportion of TFA also ranged from 0 to 33% in bakery products and from 1 to 28% in confectioneries [23, 26]. Such high variations within one food category and subgroup, reflect the current global market situation and confirm the heterogeneity of the industrial oils and fats used as well as the different uses of PHVO in food processing [27]. There appear to be variations in TFA content even between different batches of the same product.

### 4.3 Different sources and individual distribution of TFA in foods

In contrast to all the other food categories, TFA content in the 23 butter fat samples showed relative homogeneity ranging from 2.3 to 4.6% (Fig. 1). Between different countries, there is only a small variation of the TFA content in dairy products ranging from 3.2 to 6.2% [28]. In general, the sum of TFA from the recently analysed butter fat samples was rather high (3.2 ± 0.71%; median 3.0%). TFA content of ruminant derived fats (e.g. butter fat) was generally between 2 and 5%, with *t*11 being the major *trans* C18:1 isomer ranging from 30 to 70% of *trans* C18:1 (Fig. 2). The present analysis showed that TFA proportion in industrially processed foods was as high as 40%, consisting predominantly of *t*9 and *t*10 *trans* C18:1 isomers (Fig. 2). The proportion of CLA in industrially processed foods was quite low. Regarding the higher CLA proportion in ruminant fats of up to 2%, the *c*9,*t*11-CLA was the major CLA isomer (80% of total CLA by Ag^+^-HPLC, data not shown). Moreover, contents of TFA (mainly *t*11) and CLA (mainly *c*9,*t*11-CLA) in ruminant fat are increased corresponding to a high proportion of fresh grass in feed [29] confirming the exceptional position of natural produced TFA.

### 4.4 *t*9/*t*11 index

The analysis of individual *trans* C18:1 isomers is important for determining the origin of TFA in foods. The ratio of *t*9 to *t*11 (*t*9/*t*11 index) is a simple method for stating the predominance of either *t*11 or *t*9. A *t*9/*t*11 index <1 is related to ruminant fat, whereas, a *t*9/*t*11 index >1 is mostly associated with fat of industrial hydrogenation. The *t*9/*t*11 index of 0.3 is particularly low for butter fat. The low *t*9/*t*11 index at 0.5 of confectioneries containing a portion of ruminant fat reflects the higher proportion of *t*11 compared to *t*9 (Table 3). The analysis of puff pastries which are traditionally made using butter (Fig. 2) clearly exposed *t*9 as the major *trans* C18:1 isomer with a high *t*9/*t*11 index of 3.9 indicating the use of hydrogenated vegetable oils instead of pure butter fat.

Regarding the *t*9/*t*11 index in breast milk, Mueller et al. [30] demonstrated that this index reflected the portion of dairy fat in the diet. A high intake of dairy fat by the mothers, was inversely related to the *t*9/*t*11 index of breast milk. Furthermore, the dietary *t*9/*t*11 index is directly related to the *t*9/*t*11 index of the erythrocytes [31]. Thus, the *t*9/*t*11 index provides a simple, but valuable tool which should be additionally used to characterising TFA in foods and it could be used in the future for identifying the potentially health related impact of dietary lipids.

Current data does not convincingly indicate adverse effects of only R-TFA or *t*11 on human health [8, 32]. In the present study, butter fat had a mean TFA content of 3.2%, and the major *trans* isomer was *t*11 which may impart health benefits, particularly due to its conversion to *c*9,*t*11-CLA [8, 9]. Data from Motard-Bélanger et al. [7] imply that a daily dose of 1.5 en% R-TFA (4.2 g/day) does not adversely affect the lipoprotein profile.

### 4.5 Total fat and SFA content of foods

Since a high fat intake is associated with a higher risk of cardiovascular disease a daily maximum fat intake of 35 en% is recommended by the European Food Safety Authority [14].

To calculate the total TFA intake per serving, the fat content must also be taken into account. When foods have comparable TFA in relation to FAME, the total TFA in relation to one serving depends on the fat content, and can, therefore, vary enormously. For example, doughnuts from different bakery stores have a similar TFA proportion (13% of FAME), however, due to their different fat content of 15 and 32% of fresh matter, their absolute TFA content per a serving is found to be different at 1.2 g compared to 2.6 g, respectively. In the present analysis, bakery products and confectioneries contained high levels of fat (23 and 26% of fresh matter, respectively), as well as high proportions of SFA (46 and 58%, respectively). Compared to other categories, confectioneries were high in C12:0 and C14:0. This indicates the usage of palm kernel and/or coconut oil [33].

Lastly, the consumer-awareness-factor needs to be considered. In particular, unpackaged bakery products showed twofold higher TFA contents compared to packaged products. Consumers cannot distinguish between products of similar appearance having high and low TFA contents on the basis of price, smell and taste. Hence, it is altogether conceivable that within the population there are individuals or subgroups whose TFA consumption exceeds the recommended limit [20].

## 5 Conclusions

The TFA proportion in foods on the German market is declining, especially within the former high risk food groups such as French fries, margarines and shortenings. However, there is a considerable variation of TFA content, with mainly bakery products and confectioneries having extremely high values, predominantly associated with high total fat and SFA proportion. Above all, unpackaged products from unaffiliated stores had higher TFA proportions compared to packaged foods or products from fast-food chains. The total fat content of foods is also responsible for the absolute TFA content, and is consequently a significant factor to be considered. Furthermore, it is important to distinguish between the individual *trans* C18:1 as the profile usually correlates to either an industrial or a ruminant origin. Finally, in this study we have shown that the *t*9/*t*11 index can be used as an appropriate indicator to identify the source of *trans* fat.

The present food analysis forms the basis for calculating the current TFA intake in Germany's population. In general, owing to the declining trend of the TFA content in foods and by a concurrent reduction of fat intake, TFA consumption could decrease.

## Abbreviations

*t*9: elaidic acid, *t*9-C18:1
CLA: conjugated linoleic acids
FA: fatty acids
I-TFA: industrial *trans* fatty acids
C18:1: octadecenoic acid
PHVO: partially hydrogenated vegetable oils
en%: percent of energy intake
R-TFA: ruminant *trans* fatty acids
SFA: saturated fatty acids
TFA: *trans* fatty acids
*t*11: vaccenic acid, *t*11-C18:1

## References

[1] Food and Drug Administration (FDA) (2003). Department of Health and Human Services: Food labeling: *Trans* fatty acids in nutrition labeling; consumer research to consider nutrient content and health claims and possible footnote or disclosure statements; final rule and proposed rule. Fed. Regist..

[2] Craig-Schmidt MC (2006). World-wide consumption of *trans* fatty acids. Atheroscler. Suppl..

[3] Kraft J, Kuhnt K, Kramer JKG, Jahreis G (2006). *Trans*-18:1 Profile in Food and the Potential Physiological Relevance.

[4] Mensink RP, Zock PL, Kester AD, Katan MB (2003). Effects of dietary fatty acids and carbohydrates on the ratio of serum total to HDL cholesterol and on serum lipids and apolipoproteins: A meta-analysis of 60 controlled trials. Am. J. Clin. Nutr..

[5] Stender S, Astrup A, Dyerberg J (2008). Ruminant and industrially produced *trans* fatty acids: Health aspects. Food Nutr. Res..

[6] Jakobsen MU, Overvad K, Dyerberg J, Heitmann BL (2008). Intake of ruminant *trans* fatty acids and risk of coronary heart disease. Int. J. Epidemiol..

[7] Motard-Bélanger A, Charest A, Grenier G, Paquin P (2008). Study of the effect of *trans* fatty acids from ruminants on blood lipids and other risk factors for cardiovascular disease. Am. J. Clin. Nutr..

[8] Field CJ, Blewett HH, Proctor S, Vine D (2009). Human health benefits of vaccenic acid. Appl. Physiol. Nutr. Metab..

[9] Kuhnt K, Kraft J, Moeckel P, Jahreis G (2006). *Trans*-11-18:1 is effectively delta9-desaturated compared with *trans*-12-18:1 in humans. Br. J. Nutr..

[10] Hulshof KFAM, Van Erp-Baart MA, Anttolainen M, Becker W (1999). Intake of fatty acids in Western Europe with emphasis on *trans* fatty acids: The TRANSFAIR study. Eur. J. Clin. Nutr..

[11] Steinhart H, Pfalzgraf A (1992). Intake of *trans* isomeric fatty acids—an update for the Federal-Republic-of-Germany. Z. Ernahrungswiss..

[12] Fritsche J, Steinhart H (1997). Contents of *trans* fatty acids (TFA) in German foods and estimation of daily intake. Fett/Lipid.

[13] Jakobsen MU, Bysted A, Andersen NL, Heitmann BL (2006). Intake of ruminant *trans* fatty acids in the Danish population aged 1–80 years. Eur. J. Clin. Nutr..

[14] European Food Safety Authority (2010). Scientific opinion on dietary reference values for fats, including saturated fatty acids, polyunsaturated fatty acids, monounsaturated fatty acids, *trans* fatty acids, and cholesterol. EFSA Panel on Dietetic Products, Nutrition, and Allergies (NDA). EFSA J..

[15] http://www.hc-sc.gc.ca/fn-an/nutrition/gras-trans-fats/tf-ge/tf-gt_app4-eng.php.

[16] Stender S, Dyerberg J (2004). Influence of *trans* fatty acids on health. Ann. Nutr. Metab..

[17] Richter EK, Shawish KA, Scheeder MRL, Colombani PC (2009). *Trans* fatty acid content of selected Swiss foods: The Trans SwissPilot study. J. Food Comp. Anal..

[18] http://www.bmg.gv.at/cms/home/attachments/9/9/3/CH1047/CMS1291115625360/bgbla_2009_ii_267_trans-fettsauren-verordnung.pdf.

[19] Pfalzgraf A, Timm M, Steinhart H (1993). Gehalte von *trans*-Fettsäuren in Lebensmitteln. Z Ernahrungswiss..

[20] Stender S, Dyerberg J, Bysted A, Leth T, Astrup A (2006). A *trans* world journey. Atheroscler. Suppl..

[21] Folch J, Lees M, Stanley GHS (1957). A simple method for the isolation and purification of total lipids from animal tissues. J. Biol. Chem..

[22] Kuhnt K, Degen C, Jahreis G (2010). 2-Propanol in the mobile phase reduces the time of analysis of CLA isomers by silver ion-HPLC. J. Chromatogr. B.

[23] Aro A, Amaral E, Kesteloot H, Rimestad A (1998). *Trans* fatty acids in French fries, soups, and snacks from 14 European countries: The TRANSFAIR study. J. Food Comp. Anal..

[24] Dostálová J, Doležal M, Brát J, Culková J (2007). Contents of *Trans* and Other Groups of Fatty Acids in Spread Fats and Various Bakery Products.

[25] Lehner P (2007). Entwicklung der Gehalte an *trans*-Fettsäuren in ausgewählten Produkten des Österreichischen Marktes—Folgestudie der AK-Transfett-Studie 2005. Arbeitskammer Wien.

[26] Van Erp-Baart MAJ, Couet C, Cuadrado C, Kafatos A (1998). *Trans* fatty acids in bakery products from 14 European countries: The TRANSFAIR study. J. Food. Comp. Anal..

[27] Skeaff CM (2009). Feasibility of recommending certain replacement or alternative fats. Eur. J. Clin. Nutr..

[28] Aro A, Antoine JM, Pizzoferrato L, Reykdal O, Van Poppel G (1998). *Trans* fatty acids in dairy and meat products from 14 European countries: The TRANSFAIR study. J. Food. Comp. Anal..

[29] Kraft J, Collomb M, Moeckel P, Sieber R, Jahreis G (2003). Differences in CLA isomer distribution of cow's milk lipids. Lipids.

[30] Mueller A, Thijs C, Rist L, Simões-Wüst AP (2010). *Trans* fatty acids in human milk are an indicator of different maternal dietary sources containing *trans* fatty acids. Lipids.

[31] Kuhnt K, Jahreis G (2010). Selected Fatty Acids of Milk in Human Nutrition.

[32] Field AE, Willett WC, Lissner L, Colditz GA (2007). Dietary fat and weight gain among women in the Nurses' Health Study. Obesity.

[33] Dubois V, Breton S, Linder M, Fanni J, Parmentier M (2007). Fatty acid profiles of 80 vegetable oils with regard to their nutritional potential. Eur. J. Lipid Sci. Technol..

